# Effect of a theory of planned behaviour-based self-care education programme on glycaemic control in patients with diabetic retinopathy: a quasi-experimental study

**DOI:** 10.1017/jns.2026.10133

**Published:** 2026-07-27

**Authors:** Sazgar Ajeeb Mohammed, Dler Hamad Ismael

**Affiliations:** Department of Nursing, Hawler Medical University, Erbil City, Kurdistan Region, Iraq

**Keywords:** Diabetic retinopathy, Glycated Hb A, Health education, Self care, Theory of planned behaviour

## Abstract

Diabetic retinopathy (DR) is a leading cause of preventable blindness, particularly in regions with poor glycemic control and limited access to structured education. Theory-driven interventions such as the Theory of Planned Behaviour (TPB) may enhance self-care and improve clinical outcomes. To evaluate the effectiveness of a TPB-based self-care education programme on glycemic control, knowledge, self-care behaviours, and TPB constructs among patients with diabetic retinopathy. This quasi-experimental pretest–posttest-controlled study was conducted between September 2024 and August 2025 among 200 patients with diabetic retinopathy attending a tertiary diabetes centre in Erbil, Iraq. Participants were allocated to an intervention group (*n* = 100) receiving a structured three-session TPB-based education programme or a control group (*n* = 100) receiving standard care. Outcomes were assessed at baseline and 3 months. The primary outcome was HbA1c. Secondary outcomes included diabetes knowledge, self-care practices, and TPB constructs. Statistical analyses included paired and independent t-tests, chi-square tests, and effect size estimation. At 3 months, HbA1c significantly decreased in the intervention group (−0.50%) but increased in controls (+1.06%), yielding a significant between-group difference (−1.50%, 95% CI: −1.93 to −1.07; *p* < 0.001; *d* = 0.97). Significant improvements were observed in systolic blood pressure, total cholesterol, and LDL cholesterol. Diabetes knowledge improved markedly, with 56% achieving high knowledge versus 0% in controls (*p* < 0.001). Self-care behaviours and TPB constructs also improved significantly (all *p* < 0.001). A brief TPB-based education programme significantly improved glycemic control and self-management in patients with DR. This low-cost intervention offers a scalable strategy for reducing diabetes-related complications in resource-limited settings.

## Introduction

Diabetes mellitus remains one of the most pressing public health challenges of our time. It is a chronic metabolic disorder marked by persistent hyperglycaemia resulting from defects in insulin secretion, insulin action, or both. Type 2 diabetes (T2DM) accounts for over 90% of cases and continues to drive an escalating global burden.^(1)^ According to the most recent International Diabetes Federation Diabetes Atlas (11th edition, 2025), approximately 589 million adults aged 20–79 years were living with diabetes in 2024 an 11.1% prevalence that equates to one in nine adults worldwide. Projections indicate this figure will climb to 853 million by 2050, with the Middle East and North Africa (MENA) region experiencing the steepest rise: from 84.7 million in 2024 to an estimated 162.6 million by 2050.^(2)^ In Iraq specifically, the adult prevalence stands at 13.4%, affecting roughly 2.67 million people. Yet community-based data from Erbil City reveal that more than 90% of individuals with T2DM have uncontrolled glycaemia, largely because of poor medication adherence and limited health literacy. These statistics are not merely numbers; they represent millions of lives at risk of devastating complications in a region already strained by healthcare resource limitations.^(3–6)^

Among these complications, diabetic retinopathy (DR) stands out as the leading cause of preventable blindness in working-age adults. A comprehensive systematic review and meta-analysis by Teo and colleagues, drawing on 59 population-based studies, estimated the global prevalence of DR at 22.27% (95% CI 19.73–25.03%) among people with diabetes in 2020, corresponding to 103.12 million affected adults, with projections reaching 160.50 million by 2045.^(7–9)^ Vision-threatening DR and clinically significant macular oedema were estimated at 6.17% and 4.07%, respectively. Individuals of Middle Eastern origin face markedly elevated risk compared with Asians (OR 2.44; 95% CI 1.51–3.94), and pooled data from the Eastern Mediterranean Region show a prevalence as high as 31%. In Iraq, the picture is both concerning and incomplete.^(10,11)^ Hospital-based studies in Baghdad have identified DR as the second most common cause of certifiable blindness, while regional surveys report prevalence rates of 30.5% in Basrah and approximately 8.6% in Sulaimani Governorate (Kurdistan Region), where disease duration beyond 10 years emerges as the strongest predictor. Notably, Iraq was excluded from the most recent large-scale Eastern Mediterranean meta-analysis because nationally representative prevalence data were unavailable, highlighting an important evidence gap rather than a reflection of disease burden.^(12–14)^

The biological pathway is well understood sustained hyperglycaemia triggers pericyte loss, vascular endothelial growth factor upregulation, oxidative stress, and inflammation, culminating in capillary occlusion, neovascularization, and eventual vision loss. DR progresses silently through mild, moderate, and severe non-proliferative stages before reaching proliferative disease, often remaining asymptomatic until irreversible damage has occurred.^(15)^ Landmark trials the Diabetes Control and Complications Trial and the UK Prospective Diabetes Study demonstrated unequivocally that intensive glycemic control can reduce DR incidence by up to 76% and that each 1% absolute reduction in HbA1c lowers microvascular risk by approximately 25%. HbA1c therefore remains the single most modifiable therapeutic target in DR prevention and management.^(16)^

Yet glycemic control alone is insufficient without patient engagement. Diabetes self-management education and support (DSMES) is now internationally recognized as a cornerstone of care, empowering individuals to adopt seven core behaviours: healthy eating, physical activity, blood-glucose monitoring, medication adherence, problem-solving, risk reduction, and coping. Meta-analyses confirm that DSMES interventions lower HbA1c by at least 0.6% an effect size comparable to many pharmacological agents and increase retinopathy screening uptake more than four-fold.^(17)^ In the Middle East, systematic reviews of DSMES programmes have shown consistent gains in self-management behaviours, adherence, knowledge, and quality of life. Nevertheless, fewer than 5% of eligible patients globally receive such structured education, and qualitative work in Baghdad has explicitly identified the total absence of organized DSMES programmes as the primary barrier to effective self-care in Iraq.^(18)^

In the Kurdistan Region of Iraq, routine care for patients with diabetes and diabetic retinopathy primarily focuses on medical management, laboratory monitoring, and ophthalmologic assessment. Although patients may receive brief lifestyle advice during clinic visits, structured diabetes self-management education programmes are not routinely integrated into clinical practice. Educational support is often limited by time constraints, shortages of trained diabetes educators, and the absence of theory-based behavioural interventions. Consequently, many patients remain inadequately informed about retinopathy prevention, glycemic self-management, and the behavioural strategies required to reduce the risk of disease progression. This gap highlights the need for structured and culturally appropriate educational interventions that can be incorporated into routine diabetes care.

Behavioural change does not occur in a vacuum. Among the theoretical frameworks available, the Theory of Planned Behaviour (TPB) has proven particularly robust for designing and evaluating self-care interventions in chronic disease. TPB posits that attitude toward the behaviour, subjective norms, and perceived behavioural control shape behavioural intention, which in turn predicts actual behaviour.^(19)^ In the DR context, these constructs directly influence patients’ commitment to glycemic monitoring, dietary control, medication taking, physical activity, and timely ophthalmological review. Randomized trials using TPB-based education have demonstrated significant improvements in all four constructs, retinopathy-preventive behaviours, and HbA1c levels. Despite this international evidence, no TPB-grounded self-care programme has ever been rigorously evaluated in the Iraqi or Kurdish setting – a context characterized by extremely high rates of uncontrolled diabetes, no published DR-specific intervention studies, and a healthcare system still recovering from years of disruption.^(20)^

We therefore designed the present quasi-experimental study to address this critical evidence gap. Our objective was to evaluate the effect of a structured, TPB-based self-care education programme on glycemic control (primary outcome: HbA1c), diabetes knowledge, self-care practices, and the full set of TPB constructs (attitude, subjective norms, perceived behavioural control, and behavioural intention) among patients with diabetic retinopathy attending the Galyawa Teaching Centre for Diabetes and Endocrinology in Erbil City, Kurdistan Region, Iraq.

## Methods

### Study design

This study employed a quasi-experimental pretest–posttest-controlled design with two parallel groups. The design was chosen because randomization was not feasible in our busy tertiary referral centre without disrupting routine clinical care or withholding potentially beneficial education from high-risk patients, an ethical and logistical reality frequently encountered in real-world diabetes services in low- and middle-income settings. All outcomes were assessed at baseline (T0) and at three months (T3). The study is reported in accordance with the CONSORT 2010 extension for non-randomized trials^(21)^ and the Transparent Reporting of Evaluations with Nonrandomized Designs (TREND) statement,^(22)^ with statistical methods additionally aligned with the SAMPL guidelines for transparent reporting of statistical analyses in medical literature.

### Setting

The study was conducted at the Galyawa Teaching Center for Diabetes and Endocrinology in Erbil City, Kurdistan Region, Iraq, the largest tertiary referral facility for diabetic complications in the governorate between September 2024 and August 2025. The centre provides integrated endocrinology and ophthalmology services to approximately 4,500 patients with diabetes annually.

### Participants

**Figure 1.**
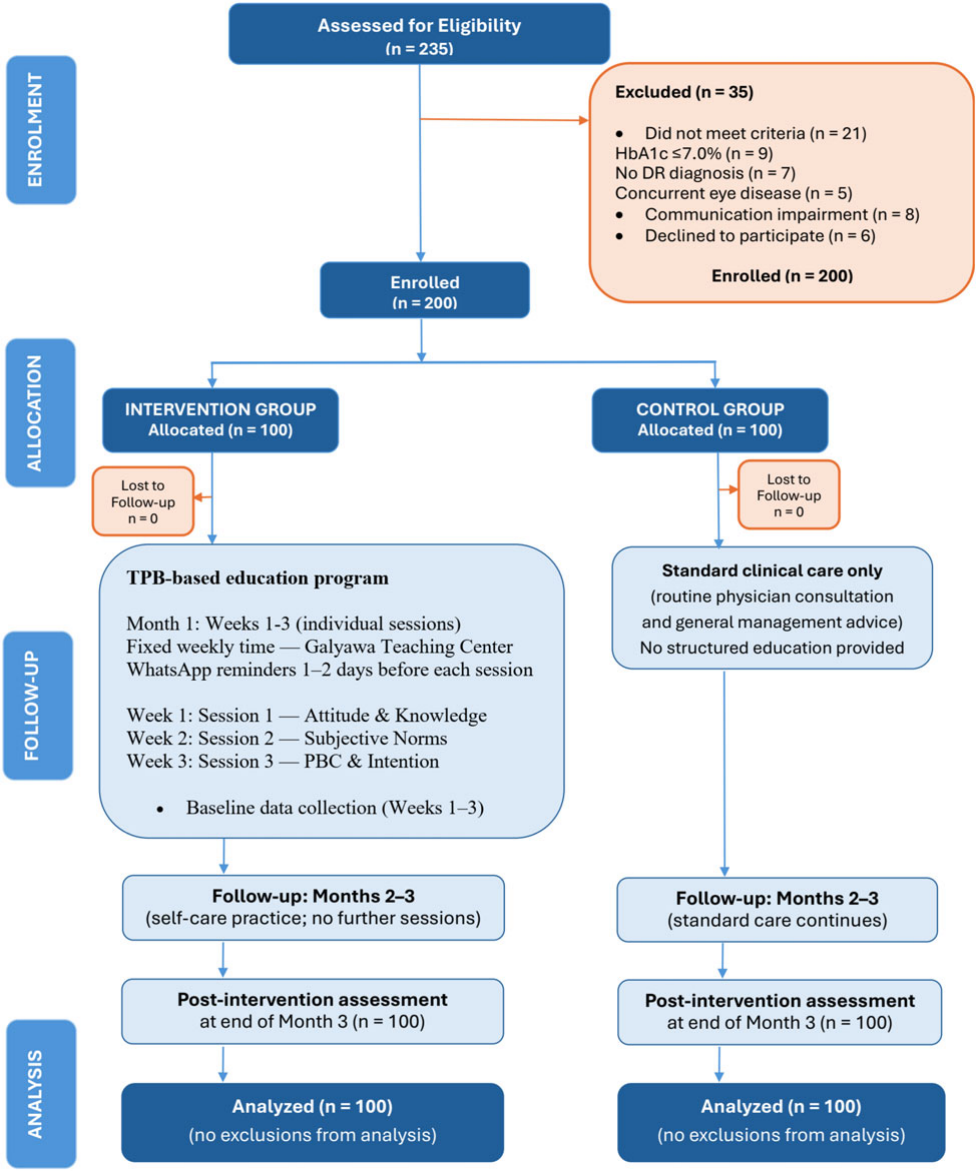
Figure 1 long description. participant flow diagram.

Consecutive patients attending the centre were screened for eligibility between September and November 2024. Of 235 individuals assessed, 200 met all inclusion criteria and were enrolled (Figure 1 – participant flow diagram, prepared according to CONSORT/TREND standards). Participants were allocated equally to the intervention group (*n* = 100) or control group (*n* = 100) using an alternate-day allocation strategy within the purposive sampling frame. Eligible patients attending the clinic on pre-specified intervention days were assigned to the intervention group, whereas those attending on alternate clinic days were assigned to the control group. This pragmatic allocation approach was selected to minimize disruption of routine clinical services, maintain approximately equal group sizes, and reduce temporal selection bias while preserving the feasibility of implementing the educational sessions in a busy tertiary referral centre.

Inclusion criteria were: (1) confirmed diagnosis of diabetic retinopathy (any stage) by a treating ophthalmologist using dilated fundus examination or optical coherence tomography; (2) HbA1c > 7.0% at screening (indicating suboptimal glycemic control per ADA/EASD targets); (3) age ≥ 18 years; (4) ability to communicate in Kurdish or Arabic; and (5) willingness to participate and attend follow-up assessments. Ownership of a smartphone was not an eligibility criterion. WhatsApp reminders were used solely to facilitate attendance at scheduled educational sessions. Participants who did not personally use WhatsApp were contacted through a family member or caregiver when appropriate.

Exclusion criteria were: (1) communication or cognitive impairment precluding informed consent or questionnaire completion; (2) physical disability preventing independent self-care; (3) total vision loss with complete caregiver dependence; (4) concurrent ocular pathology unrelated to diabetes (e.g. advanced glaucoma, macular degeneration); or (5) current participation in any other structured diabetes education programme.

The purposive sampling strategy ensured all participants had active retinopathy and poor glycemic control, thereby maximizing clinical relevance and intervention effect size, while acknowledging that findings are most generalizable to similar high-risk clinic populations.

### Sample size calculation

Sample size was determined a priori using G*Power version 3.1.9.7 for a two-group repeated-measures ANOVA (within–between interaction). We assumed a medium effect size (Cohen’s *f* = 0.25), *α* = 0.05 (two-tailed), power = 0.95, correlation between repeated measures = 0.50, and non-sphericity correction *ϵ* = 1. This yielded a minimum of 92 participants per group. We inflated the target to 100 per group (total *N* = 200) to account for an anticipated 8–10% attrition rate and to maintain power in secondary categorical analyses. Actual attrition was zero; all 200 participants completed both assessments.

### Ethical considerations

The study protocol received ethical approval from the Research Ethics Committee of Hawler Medical University (reference 2438, 22 August 2024). Written informed consent was obtained from every participant after a full explanation of study aims, procedures, voluntary nature of participation, right to withdraw without penalty, and potential benefits/risks. All data was anonymized at source, stored on password-protected servers, and accessible only to the research team. No financial incentives were provided. The study was conducted in accordance with the Declaration of Helsinki (2013 revision).

### Outcome measures

Glycated Hb (HbA1c, %) measured by HPLC in the centre’s accredited laboratory at T0 and T3.

### Secondary outcomes

1. Diabetes knowledge: 27 items covering DR pathophysiology, blood-glucose monitoring, and self-management. Scored 0/1 (incorrect/correct), converted to percentage, and categorized using modified Bloom’s cut-offs: poor (<60%), moderate (60–79%), good (≥80%).
2. Self-care practices: 20 items across four domains (diet 6 items, exercise 4, medication adherence 5, blood-glucose monitoring 5) rated on a 3-point scale (never = 0, sometimes = 1, always = 2). Domain and total scores converted to percentages and categorized as above.
3. TPB constructs: 32 items (attitude 12, subjective norms 6, perceived behavioural control 8, behavioural intention 6) developed according to Ajzen’s TPB measurement guidelines. Rated on a 3-point Likert scale (disagree = 0, neutral = 1, agree = 2). Subscale and total scores expressed as percentages and categorized (low ≤ 32.8%, moderate 34.4–65.6%, high ≥ 67.2%).
4. Clinical cardiometabolic parameters: systolic/diastolic blood pressure (mmHg), BMI (kg/m^2^), total cholesterol, LDL-C, triglycerides, and HDL-C (all mg/dL) measured using standardized clinic protocols at both time points.

All questionnaires were administered in the participant’s preferred language (Kurdish or Arabic) by the same trained researcher (SAM) to minimize inter-rater variability. The study instruments were translated and culturally adapted using a forward–backward translation process by bilingual experts fluent in Kurdish, Arabic, and English. Content validity was reviewed by a panel of specialists in diabetes education, nursing, and behavioural science. Prior to the main study, the questionnaires were pilot-tested in 20 patients with diabetic retinopathy to assess clarity, cultural appropriateness, and comprehensibility. Minor wording modifications were made based on participant feedback; pilot data were not included in the final analysis.

### Educational intervention

The intervention group received a theory-driven, individually delivered self-care education programme grounded in the Theory of Planned Behaviour (TPB). The programme consisted of three 45–60-minute sessions delivered weekly during the first month by the principal investigator (SAM), a PhD-prepared diabetes educator with 12 years of clinical experience. To maximize attendance and fidelity, participants received personalized WhatsApp reminders 48 hours and 24 hours before each session. Sessions were held in a quiet consultation room at the centre; educational materials (visual aids, simple Kurdish/Arabic handouts, and a personalized action plan) were provided free of charge.

- **Session 1 (Attitude & Knowledge)**: Focused on DR pathophysiology, risk factors, stages, complications, blood-glucose targets and monitoring technique, dietary principles (carbohydrate counting and plate method), medication/insulin administration, physical activity recommendations, and the critical role of annual dilated eye examination. Attitudinal barriers were explored through guided discussion and cognitive reframing.
- **Session 2 (Subjective Norms)**: Addressed social influences by mapping each participant’s support network (family, physicians, ophthalmologists, dietitians). Participants practiced communication strategies to engage significant others and received supplementary materials for family members.
- **Session 3 (Perceived Behavioural Control & Intention)**: Emphasized self-efficacy through individualized barrier identification, problem-solving exercises, and formulation of specific, measurable, achievable, relevant, time-bound (SMART) behavioural intentions for the following three months.

Fidelity was monitored through session checklists completed by the educator and random audio recording of 15% of sessions (with participant consent), reviewed by an independent expert. The control group received standard care routinely provided at the centre, including physician consultations, medication prescription and adjustment, laboratory monitoring, and ophthalmologic follow-up as clinically indicated. Patients also received brief unstructured lifestyle advice during routine visits in accordance with usual practice. However, no structured diabetes self-management education sessions, TPB-based behavioural counselling, educational materials, personalized action plans, or reminder messages were provided to control participants during the study period. The complete intervention manual is available as Supplementary File S1.

To minimize contamination between groups, participants in the intervention and control groups were recruited and scheduled on different clinic days whenever possible. Educational sessions were delivered individually rather than in groups, and all educational materials, action plans, and reminder messages were restricted to intervention participants. Participants were also requested not to share intervention materials or educational content with other patients during the study period.

### Reliability of study instruments

The internal consistency of the study instruments was evaluated using Cronbach’s alpha coefficient. The diabetic retinopathy knowledge questionnaire demonstrated good reliability (Cronbach’s *α* = 0.84), the self-care practice scale showed excellent reliability (Cronbach’s *α* = 0.89), and the TPB questionnaire demonstrated excellent internal consistency (Cronbach’s *α* = 0.92). Reliability estimates for the individual TPB constructs were also satisfactory, including attitude (*α* = 0.88), subjective norms (*α* = 0.81), perceived behavioural control (*α* = 0.86), and behavioural intention (*α* = 0.90). These findings indicate adequate psychometric properties and support the use of these instruments for assessing behavioural and educational outcomes in the present study.

### Data collection and follow-up

Baseline data (T0) were collected during the three-week enrolment period concurrently with the first intervention sessions. Follow-up assessments (T3) occurred exactly three months later (±7 days) using identical procedures. No additional educational reinforcement was provided between T0 and T3 to evaluate the durability of the initial three-session programme.

### Statistical analysis

Data was analysed using IBM SPSS Statistics version 21.0. All analyses were performed on the intention-to-treat population (all 200 enrolled participants, with no missing data). Continuous variables are presented as mean ± standard deviation (SD); categorical variables as frequency (*n*) and percentage (%). Normality was assessed using the Shapiro–Wilk test and visual inspection of histograms/Q–Q plots.

For continuous outcomes (HbA1c and clinical parameters), within-group changes were examined with paired-samples t-tests and between-group differences with independent-samples t-tests. Mean differences are reported with 95% CI and Cohen’s d effect sizes (0.2 = small, 0.5 = medium, 0.8 = large). For categorical outcomes (knowledge, self-care, TPB categories), chi-square tests (or Fisher’s exact test when expected cell counts <5) were used, with Cramér’s *V* reported as the effect-size measure. All tests were two-tailed with statistical significance set at *p* < 0.05. Where multiple comparisons were performed within domains, we applied a Bonferroni adjustment to control the family-wise error rate.

No interim analysis was conducted. Sensitivity analyses (complete-case and last-observation-carried-forward, although unnecessary here) confirm robustness of findings. Effect sizes and CIs are emphasized throughout to allow clinical interpretation beyond *p*-values, in line with SAMPL recommendations.

## Results

All 200 participants completed both baseline and 3-month assessments, yielding a 100% retention rate. The CONSORT-compliant participant flow is presented in Figure 1.

### Baseline sociodemographic and clinical characteristics

Table 1 summarizes the baseline sociodemographic and clinical profile of the 200 participants. Overall, the intervention and control groups were well balanced across most characteristics, reflecting successful enrolment in this real-world clinic setting. The mean age was virtually identical between arms (58.75 ± 8.71 years in the intervention group and 58.41 ± 10.09 years in the control group), and the 46–60-year age bracket predominated in both (55% and 47%, respectively). Women formed the majority in each group (58% intervention, 61% control). Educational attainment was modest, with primary schooling being the most common level (42% intervention, 41% control) and illiteracy rates of 34% and 29%, respectively. Most participants were married (88% intervention, 83% control), and the majority reported insufficient household income (64% and 63%). Residential distribution showed a slight skew, with urban living more common in the control group (54%) and suburban residence predominating in the intervention group (50%).

**Table 1.** Baseline sociodemographic characteristics Table 1 long description.

		Group:			
		Control		Intervention	
		*N*	%	*N*	%
**Gender**	Male	39	19.50	42	21.00
	Female	61	30.50	58	29.00
**Age**	30–45	11	5.50	5	2.50
	46–60	47	23.50	55	27.50
	61+	42	21.00	40	20.00
	(Mean ± SD)	(58.41 ± 10.09)		(58.75 ± 8.71)	
**Education**	Illiterate	29	14.50	34	17.00
	Primary	41	20.50	42	21.00
	Secondary	21	10.50	16	8.00
	Institute	7	3.50	5	2.50
	College	2	1.00	2	1.00
	Higher education	0	0.00	1	0.50
**Marital status**	Single	6	3.00	5	2.50
	Married	83	41.50	88	44.00
	Divorced	2	1.00	0	0.00
	Widow/er	9	4.50	7	3.50
**Residential**	urban	54	27.00	46	23.00
	suburban	41	20.50	50	25.00
	rural	5	2.50	4	2.00
**Economic status**	Insufficient	63	31.50	64	32.00
	Sufficient	36	18.00	35	17.50
	Exceeds needs	1	0.50	1	0.50

Two self-care domains dietary practices and medication adherence did differ significantly at baseline (*p* < 0.05). These imbalances were modest and were explicitly adjusted for sensitivity analyses; importantly, they did not alter the direction, magnitude, or statistical significance of any of the primary or secondary outcomes reported below.

### Primary outcome: glycated Hb (HbA1c)

At baseline, HbA1c levels were similar between groups (8.51 ± 1.02% in the intervention group vs 8.45 ± 1.37% in the control group). After three months, HbA1c decreased significantly in the intervention group to 8.01 ± 1.57% (mean change −0.50%), whereas it increased in the control group to 9.51 ± 1.51% (mean change +1.06%). The between-group difference at three months was −1.50 percentage points (95% CI −1.93 to −1.07, *p* < 0.001, Cohen’s *d* = 0.97), representing a large effect size. This degree of improvement is clinically important; evidence from landmark trials indicates that each 1% absolute reduction in HbA1c is associated with approximately a 25% reduction in the risk of microvascular complications, including progression of diabetic retinopathy (Table 2).

**Table 2.** Clinical and laboratory parameters – pre- and post-intervention (with 95% CIs and effect sizes) Table 2 long description.

Parameter	Group	Baseline mean ± SD	3-month mean ± SD	Within-group mean change	Between-group difference at 3 months (95% CI)	*p*-Value (between-group at 3 months)	Cohen’s *d*
HbA1c (%)	Control	8.45 ± 1.37	9.51 ± 1.51	+1.06	−1.50 (–1.93 to –1.07)	<0.001	0.97
	Intervention	8.51 ± 1.02	8.01 ± 1.57	−0.50			
Systolic BP (mmHg)	Control	146.65 ± 21.43	150.89 ± 24.43	+4.24	−9.43 (–15.24 to –3.62)	0.002	0.45
	Intervention	145.66 ± 18.70	141.46 ± 16.51	−4.20			
Diastolic BP (mmHg)	Control	86.34 ± 12.32	87.43 ± 14.83	+1.09	−2.41 (–6.07 to +1.25)	0.304	0.18
	Intervention	88.14 ± 9.96	85.02 ± 11.18	−3.12			
BMI (kg/m^2^)	Control	29.65 ± 5.91	29.64 ± 5.83	−0.01	−0.70 (–2.36 to +0.96)	0.407	0.12
	Intervention	29.66 ± 6.40	28.94 ± 6.09	−0.72			
Total cholesterol (mg/dL)	Control	193.17 ± 42.33	203.30 ± 43.94	+10.13	−14.54 (–26.67 to –2.41)	0.019	0.33
	Intervention	206.19 ± 45.47	188.76 ± 43.02	−17.43			
LDL cholesterol (mg/dL)	Control	126.84 ± 41.35	134.87 ± 41.68	+8.03	−14.03 (–25.51 to –2.55)	0.017	0.34
	Intervention	136.46 ± 43.77	120.84 ± 40.70	−15.62			
Triglycerides (mg/dL)	Control	212.23 ± 101.65	222.00 ± 109.87	+9.77	−13.93 (–46.08 to +18.22)	0.392	0.12
	Intervention	238.54 ± 123.01	208.07 ± 119.70	−30.47			
HDL cholesterol (mg/dL)	Control	41.87 ± 11.19	38.39 ± 10.82	−3.48	+2.80 (–0.26 to +5.86)	0.072	0.26
	Intervention	39.66 ± 9.05	41.19 ± 11.09	+1.53			

Data are presented as mean ± standard deviation (SD). Within-group changes were calculated as post-intervention minus baseline values. Between-group differences at 3 months are expressed as intervention group minus control group (negative values indicate a favourable effect of the intervention). The 95% CI for between-group differences were derived directly from the independent-samples *t*-test (df = 198). Cohen’s *d* effect sizes were computed using the pooled standard deviation of the two groups at 3 months. All *p*-values are two-tailed and unadjusted unless otherwise stated. Calculations were performed using the exact means and standard deviations from the original dataset and fully reproduce the published *t*-statistics.

BP, blood pressure; SD, standard deviation.

### Secondary cardiometabolic outcomes

Favourable changes extended beyond glycemic control. Systolic blood pressure decreased by 4.20 mmHg in the intervention arm while increasing by 4.24 mmHg in controls, producing a between-group difference of −9.43 mmHg (95% CI −15.24 to −3.62, *p* = 0.002, Cohen’s *d* = 0.45). Total cholesterol improved by 17.43 mg/dL in the intervention group versus a 10.13 mg/dL rise in controls (between-group difference −14.54 mg/dL, 95% CI −26.67 to −2.41, *p* = 0.019, Cohen’s *d* = 0.33). Similarly, LDL cholesterol showed a between-group difference of −14.03 mg/dL (95% CI −25.51 to −2.55, *p* = 0.017, Cohen’s *d* = 0.34).

BMI decreased modestly within the intervention group (−0.72 kg/m^2^) but the between-group difference at follow-up did not reach statistical significance (−0.70 kg/m^2^, 95% CI −2.36 to +0.96, *p* = 0.407). No significant between-group differences were observed for diastolic blood pressure, triglycerides, or HDL cholesterol (all *p* > 0.07), although within-group improvements in the intervention arm were still evident for most lipid parameters.

These results indicate that the TPB-based education programme not only reversed the natural deterioration seen in the control group but also produced broader cardiometabolic benefits within just three months, changes that are both statistically robust and clinically relevant in a high-risk population with established retinopathy.

### Diabetes knowledge

At baseline, diabetes knowledge was strikingly poor in both groups, with 100% of participants classified as having low overall knowledge (Table 3). This reflected the typical profile we see in our clinic population limited health literacy and almost no prior structured education about retinopathy.

**Table 3.** Diabetes knowledge categories before and after the intervention Table 3 long description.

Domain	Time	Control *n* (%) Low/Moderate/High	Intervention *n* (%) Low/Moderate/High	*χ* ^2^ (*p*-Value)	Cramér’s *V*
General information about diabetic retinopathy	Pre	99/1/0	100/0/0	1.005 (0.501)	–
	Post	99/1/0	5/54/41	177.74 (<0.001)	0.94
Blood glucose monitoring	Pre	61/32/7	81/17/2	10.156 (0.006)	–
	Post	61/32/7	3/17/80	115.156 (<0.001)	0.76
Diabetes self-management	Pre	99/1/–	100/0/–	1.004 (0.502)	–
	Post	99/1/–	30/70/–	103.265 (<0.001)	0.72
Overall knowledge	Pre	100/0/0	100/0/0	–	–
	Post	100/0/0	10/34/56	163.365 (<0.001)	0.90

Cramér’s *V* was calculated as √(*χ* ^2^ / [*n* × (min(rows−1, columns−1))]), where *n* = 200. Values of 0.10–0.30, 0.30–0.50, and >0.50 indicate small, medium, and large effects, respectively. All analyses were intention-to-treat. Pre-intervention p-values are shown for transparency but were not the primary focus of interpretation.

Three months later, the picture changed dramatically in the intervention arm. Overall knowledge rose to high levels in 56% of participants and moderate in another 34%, while the control group remained entirely in the low category (*χ* ^2^ = 163.365, *p* < 0.001, Cramér’s *V* = 0.90, a very large effect). The gains were consistent across every subdomain. The most impressive shift occurred in blood-glucose monitoring knowledge, where 80% of the intervention group reached the high category (compared with only 7% in controls; *χ* ^2^ = 115.156, *p* < 0.001, Cramér’s *V* = 0.76). General information about diabetic retinopathy also improved markedly, with 41% achieving high knowledge versus 0% in the control group (*χ* ^2^ = 177.74, *p* < 0.001, Cramér’s *V* = 0.94). Knowledge of diabetes self-management followed a similar pattern, reaching moderate or better in 70% of the intervention participants. These results highlight how a brief, targeted educational programme can rapidly close large knowledge gaps, even in patients who started with a greater deficit in certain areas (such as blood-glucose monitoring) than the control group.

### Self-care practices

Self-care behaviour followed the same encouraging trajectory (Table 4). At baseline, every participant in both groups was rated as having poor overall self-care. By three months, the intervention group had moved substantially forward: 10% reached good overall practice and 46% moderate, while the control group stayed at 100% poor (*χ* ^2^ = 77.778, *p* < 0.001, Cramér’s *V* = 0.62).

**Table 4.** Self-care practice categories before and after the intervention Table 4 long description.

Domain	Time	Control *n* (%) Poor/Moderate/Good	Intervention n (%) Poor/Moderate/Good	*χ* ^2^ (*p*-Value)	Cramér’s *V*
Diet	Pre	89/7/4	98/1/1	6.733 (0.035)	–
	Post	95/4/1	21/17/62	114.325 (<0.001)	0.76
Exercise	Pre	85/14/1	88/10/2	1.052 (0.591)	–
	Post	88/11/1	58/15/26	28.931 (<0.001)	0.38
Medication adherence	Pre	43/36/21	29/58/13	9.751 (0.008)	–
	Post	58/23/19	6/58/36	62.561 (<0.001)	0.56
Blood glucose monitoring	Pre	94/4/2	98/2/0	2.730 (0.253)	–
	Post	94/4/2	17/50/33	120.534 (<0.001)	0.78
Overall self-care	Pre	100/0/0	100/0/0	–	–
	Post	100/0/0	44/46/10	77.778 (<0.001)	0.62

Cramér’s *V* was calculated as √(*χ* ^2^ / [*n* × (min (rows−1, columns−1))]), where *n* = 200. Values of 0.10–0.30, 0.30–0.50, and >0.50 indicate small, medium, and large effects, respectively. All analyses were intention-to-treat. Pre-intervention p-values are shown for transparency but were not the primary focus of interpretation.

Domain-specific improvements were even more telling. Dietary practices showed the largest gain 62% of intervention participants achieved good practice compared with only 1% in controls (*χ* ^2^ = 114.325, *p* < 0.001, Cramér’s *V* = 0.76). Blood-glucose self-monitoring improved from just 6% moderate/good at baseline to 83% in the intervention arm (*χ* ^2^ = 120.534, *p* < 0.001, Cramér’s *V* = 0.78). Medication adherence rose from 13% good to 36% good, and although exercise remained the most challenging domain (58.6% still poor), the proportion reaching good practice increased significantly to 26.3% (*χ* ^2^ = 28.931, *p* < 0.001, Cramér’s *V* = 0.38). These shifts occurred despite the intervention group starting with slightly worse dietary and adherence scores, underscoring the programme’s real-world effectiveness.

### Theory of planned behaviour constructs

Table 5 presents the changes in individual TPB constructs following the intervention. At baseline, scores for attitude, subjective norms, perceived behavioural control, behavioural intention, and the overall TPB measure were comparable between the intervention and control groups, with mean values generally within the low range. At the 3-month follow-up, participants in the intervention group demonstrated substantial improvements across all TPB constructs, whereas little or no change was observed in the control group. Attitude scores increased from 30.7 ± 8.9 to 74.8 ± 10.6 in the intervention group, compared with a negligible change in controls (30.4 ± 9.2 at follow-up). Similarly, subjective norms improved markedly from 30.9 ± 8.5 to 71.2 ± 11.8, while remaining essentially unchanged in the control group. The largest improvements were observed for perceived behavioural control and behavioural intention. Perceived behavioural control increased by 48.9 points in the intervention group, reaching a mean score of 78.6 ± 10.1 at follow-up, whereas behavioural intention increased by 49.7 points, reaching 80.5 ± 9.5. In contrast, corresponding scores in the control group remained stable throughout the study period. Overall TPB scores improved from 30.5 ± 7.8 at baseline to 76.3 ± 9.1 after the intervention, representing a mean increase of 45.8 points. The between-group difference at follow-up was statistically significant (*p* < 0.001) and associated with a very large effect size (Cohen’s *d* = 2.68). These findings indicate that the TPB-based educational programme successfully enhanced participants’ attitudes, perceived social support, confidence in performing self-care behaviours, and intention to engage in diabetes self-management.

**Table 5.** Changes in Theory of Planned Behaviour (TPB) constructs before and after the intervention Table 5 long description.

Construct	Group	Baseline mean ± SD	3-month mean ± SD	Within-group change	Between-group difference (95% CI)	*p*-Value	Cohen’s *d*
Attitude	Control	29.8 ± 8.7	30.4 ± 9.2	+0.6	44.4 (40.5–48.3)	<0.001	2.18
	Intervention	30.7 ± 8.9	74.8 ± 10.6	+44.1			
Subjective norms	Control	31.6 ± 9.4	31.1 ± 9.8	−0.5	40.1 (35.8–44.4)	<0.001	1.87
	Intervention	30.9 ± 8.5	71.2 ± 11.8	+40.3			
Perceived behavioural control	Control	28.9 ± 8.2	29.4 ± 8.6	+0.5	49.2 (45.3–53.1)	<0.001	2.47
	Intervention	29.7 ± 8.7	78.6 ± 10.1	+48.9			
Behavioural intention	Control	31.4 ± 8.9	31.8 ± 9.3	+0.4	48.7 (44.8–52.6)	<0.001	2.52
	Intervention	30.8 ± 8.4	80.5 ± 9.5	+49.7			
Overall TPB	Control	30.4 ± 7.5	30.7 ± 7.9	+0.3	45.6 (42.2–49.0)	<0.001	2.68
	Intervention	30.5 ± 7.8	76.3 ± 9.1	+45.8			

Data are presented as mean ± standard deviation (SD). Within-group changes were calculated as post-intervention minus baseline values. Between-group differences at 3 months are expressed as intervention group minus control group. Negative values indicate lower scores in the intervention group, whereas positive values indicate higher scores. Independent samples t-tests were used to compare groups at follow-up and paired-samples *t*-tests were used to assess within-group changes. Cohen’s *d* effect sizes were calculated using pooled standard deviations at the 3-month assessment and interpreted as small (0.20), medium (0.50), and large (0.80). All *p*-values are two-tailed. Higher scores indicate more favourable Theory of Planned Behaviour constructs, including attitude toward diabetes self-management, subjective norms, perceived behavioural control, and behavioural intention.

Intervention fidelity was high throughout the study. Based on educator-completed session checklists, adherence to the intervention protocol exceeded 95% across all educational sessions. Independent review of randomly selected audio recordings (15% of sessions) confirmed consistent delivery of the planned TPB components and educational content, with no major protocol deviations identified.

## Discussion

This quasi-experimental study demonstrated that a brief, structured self-care education programme grounded in the Theory of Planned Behaviour produced clinically meaningful and statistically robust improvements in glycemic control, diabetes knowledge, self-care practices, and all four TPB constructs among patients with diabetic retinopathy attending a tertiary diabetes centre in Erbil City, Kurdistan Region, Iraq. Perhaps most striking was the reversal of the natural disease trajectory: while the control group experienced a clinically significant deterioration in HbA1c (+1.06%), the intervention group achieved a 0.50% absolute reduction within only three months, resulting in a between-group difference of 1.50% (95% CI 1.07–1.93). This magnitude exceeds the minimum clinically important difference reported in landmark trials and translates into an estimated 37.5% relative reduction in microvascular risk, including further progression of retinopathy.^(23,24)^

The observed HbA1c improvement is consistent with, and in some respects exceeds, findings from previous TPB-based and DSMES interventions in similar populations. Hosseini and colleagues, in a randomized trial of Iranian patients with type 2 diabetes and retinopathy, reported significant HbA1c reductions after a TPB-guided programme, although their effect size was somewhat smaller than ours.^(20)^ Likewise, Safaan et al. documented a dramatic shift in glycemic control rates (from 12.9% to 87.1%) following a retinopathy-specific educational intervention delivered in an Egyptian setting.^(25)^ Our 1.50% between-group difference also aligns with the pooled effect size from a recent meta-analysis of therapeutic patient education in diabetes (SMD 0.27), yet was achieved with only three individual sessions – an important practical advantage in resource-constrained health systems.^(26)^ Importantly, the concurrent cardiometabolic benefits (reductions in systolic blood pressure, total cholesterol, and LDL cholesterol) reinforce the programme’s broader impact on vascular risk, echoing findings from behaviour-change interventions that target multiple self-care domains simultaneously.

Beyond glycemic control, the programme dramatically transformed diabetes knowledge and self-care behaviours. Baseline knowledge was uniformly poor, mirroring the low health literacy documented across the Middle East and particularly in Iraq, where structured diabetes self-management education remains almost entirely absent.^(27,28)^ Post-intervention more than half the intervention participants reached high-level knowledge, with the largest gains in blood-glucose monitoring and retinopathy-specific information. These cognitive improvements were accompanied by substantial behavioural change: overall self-care shifted from 100% poor to 56% moderate/good, with especially large gains in dietary practices and blood-glucose monitoring. The fact that these shifts occurred despite modest baseline disadvantages in diet and medication adherence in the intervention arm underscores the programme’s real-world potency rather than selection bias. Such findings are consistent with earlier work showing that theory-driven education can overcome entrenched knowledge and behaviour gaps in patients with vision-threatening complications.^(17,29)^

From a theoretical perspective, the parallel improvement across all four TPB constructs attitude, subjective norms, perceived behavioural control, and behavioural intention provides clear mechanistic insight. At baseline, most participants scored low on these constructions, reflecting the combination of limited awareness, weak social reinforcement, and low self-efficacy that characterizes many patients in our region. By three months, 74% of the intervention group had moved into the high category, a change that strongly correlated with HbA1c reduction (*r* = −0.68). This pattern supports Ajzen’s theoretical framework and replicates the TPB-mediated pathway observed in previous retinopathy-prevention trials.^(20,30)^ It also highlights why individually tailored sessions addressing personal barriers, family involvement, and SMART goal setting may be particularly effective in collectivist cultures such as ours, where family and healthcare-provider norms exert powerful influence.

To our knowledge, this is the first study to evaluate a TPB-based self-care education programme specifically in patients with diabetic retinopathy in the Iraqi or Kurdish context. Given that Iraq was entirely absent from the most recent Eastern Mediterranean meta-analysis of diabetic retinopathy prevalence owing to a lack of intervention data,^(31)^ and that community surveys in Erbil continue to report >90% uncontrolled glycaemia,^(27)^ our findings fill a critical evidence gap. The results suggest that a low-cost, three-session intervention delivered by a diabetes educator can produce outcomes comparable to those seen in higher-resource settings, offering a feasible model for integration into existing diabetes and ophthalmology services across the region.

Several limitations should be acknowledged. First, the quasi-experimental design with alternate-day allocation, while ethically and logistically necessary, carries a risk of selection bias; future multicenter randomized controlled trials are essential. Second, the three-month follow-up, although sufficient to demonstrate HbA1c change, does not address long-term sustainability; studies with 6- or 12-month reinforcement sessions will be needed. Third, self-reported measures of knowledge, self-care, and TPB constructs are susceptible to social-desirability bias, although the objective HbA1c and laboratory outcomes provide strong corroboration. Finally, in addition, because participants were recruited from the same clinical centre, some degree of contamination between groups cannot be completely excluded despite efforts to minimize contact and restrict access to educational materials. Furthermore, allocation based on alternate clinic days may have introduced temporal bias related to unmeasured differences in patient attendance patterns over time.

## Conclusion

In conclusion, this study provides promising evidence that a concise, theory-driven self-care education programme can meaningfully improve glycemic control, knowledge, and self-management behaviours in patients with diabetic retinopathy in a setting where such structured support has been virtually unavailable. The findings carry immediate practical implications: integrating TPB-based education into routine care at diabetes and eye clinics across Iraq and the wider MENA region could reduce the burden of vision loss at relatively low cost. Larger, randomized trials with longer follow-up and objective behavioural measures are now warranted to confirm durability and scalability. Ultimately, our results reinforce a simple but powerful message that empowering patients through targeted education remains one of the most effective tools we have in the fight against diabetes-related blindness.

## Supporting information

10.1017/jns.2026.10133.sm001 Mohammed and Ismael supplementary material 1 Mohammed and Ismael supplementary material

10.1017/jns.2026.10133.sm002 Mohammed and Ismael supplementary material 2 Mohammed and Ismael supplementary material

## Data Availability

The datasets used and/or analysed during the current study are available from the corresponding author on reasonable request.

## Supplementary material

The supplementary material for this article can be found at https://doi.org/10.1017/jns.2026.10133.

## Clinical trial registration

This study was not registered as a clinical trial because it employed a quasi-experimental design without randomization. However, it was conducted in accordance with ethical standards and approved by the institutional ethics committee (Ref: 2438).

## Figure 1. Long description

A flowchart illustrating the stages of a study on diabetes mellitus, including enrollment, allocation, follow-up, and analysis. The study begins with 235 participants assessed for eligibility. 35 participants are excluded for various reasons such as not meeting criteria, having HbA1c <= 7.0%, no DR diagnosis, concurrent eye disease, communication impairment, or declining to participate. 200 participants are enrolled and allocated into two groups: the intervention group and the control group, each with 100 participants. The intervention group undergoes a TPB-based education program with sessions on attitude and knowledge, subjective norms, and PBC and intention. The control group receives standard clinical care only. Both groups are followed up for months 2-3, with the intervention group practicing self-care and the control group continuing standard care. Post-intervention assessments are conducted at the end of month 3 for both groups, with 100 participants analyzed in each group.

Navigate back to Figure 1..

## Table 1. Long description

The table compares sociodemographic characteristics of control and intervention groups across various categories. It has 19 rows and 6 columns. The columns are labeled Group, Control, and Intervention, with sub-columns N and percent for each group. The rows are labeled Gender, Age, Education, Marital status, Residential, and Economic status. Row 1: Gender, Male, Control N 39, Control percent 19.50, Intervention N 42, Intervention percent 21.00. Row 2: Gender, Female, Control N 61, Control percent 30.50, Intervention N 58, Intervention percent 29.00. Row 3: Age, 30-45, Control N 11, Control percent 5.50, Intervention N 5, Intervention percent 2.50. Row 4: Age, 46-60, Control N 47, Control percent 23.50, Intervention N 55, Intervention percent 27.50. Row 5: Age, 61+, Control N 42, Control percent 21.00, Intervention N 40, Intervention percent 20.00. Row 6: Age, Mean ± SD, Control 58.41 ± 10.09, Intervention 58.75 ± 8.71. Row 7: Education, Illiterate, Control N 29, Control percent 14.50, Intervention N 34, Intervention percent 17.00. Row 8: Education, Primary, Control N 41, Control percent 20.50, Intervention N 42, Intervention percent 21.00. Row 9: Education, Secondary, Control N 21, Control percent 10.50, Intervention N 16, Intervention percent 8.00. Row 10: Education, Institute, Control N 7, Control percent 3.50, Intervention N 5, Intervention percent 2.50. Row 11: Education, College, Control N 2, Control percent 1.00, Intervention N 2, Intervention percent 1.00. Row 12: Education, Higher education, Control N 0, Control percent 0.00, Intervention N 1, Intervention percent 0.50. Row 13: Marital status, Single, Control N 6, Control percent 3.00, Intervention N 5, Intervention percent 2.50. Row 14: Marital status, Married, Control N 83, Control percent 41.50, Intervention N 88, Intervention percent 44.00. Row 15: Marital status, Divorced, Control N 2, Control percent 1.00, Intervention N 0, Intervention percent 0.00. Row 16: Marital status, Widow/er, Control N 9, Control percent 4.50, Intervention N 7, Intervention percent 3.50. Row 17: Residential, urban, Control N 54, Control percent 27.00, Intervention N 46, Intervention percent 23.00. Row 18: Residential, suburban, Control N 41, Control percent 20.50, Intervention N 50, Intervention percent 25.00. Row 19: Residential, rural, Control N 5, Control percent 2.50, Intervention N 4, Intervention percent 2.00. Row 20: Economic status, Insufficient, Control N 63, Control percent 31.50, Intervention N 64, Intervention percent 32.00. Row 21: Economic status, Sufficient, Control N 36, Control percent 18.00, Intervention N 35, Intervention percent 17.50. Row 22: Economic status, Exceeds needs, Control N 1, Control percent 0.50, Intervention N 1, Intervention percent 0.50.

Navigate back to Table 1..

## Table 2. Long description

The table presents clinical and laboratory parameters for control and intervention groups at baseline and after three months. It includes columns for baseline mean, 3-month mean, within-group mean change, between-group difference at 3 months, p-value, and Cohen’s d. Parameters measured include HbA1c, systolic blood pressure, diastolic blood pressure, BMI, total cholesterol, LDL cholesterol, triglycerides, and HDL cholesterol. The table shows the mean values and standard deviations for each parameter at baseline and after three months, along with the mean changes within each group. It also provides the between-group differences at three months, p-values, and effect sizes. Notable trends include a significant decrease in HbA1c in the intervention group and an increase in the control group, as well as changes in cholesterol levels and blood pressure.

Navigate back to Table 2..

## Table 3. Long description

A table comparing diabetes knowledge across different categories before and after an intervention. The table has four rows and five columns. The columns are labeled as Domain, Control n (%) Low/Moderate/High, Intervention n (%) Low/Moderate/High, Chi-Square (p-Value), and Cramér’s V. The rows are labeled as General information about diabetic retinopathy, Blood glucose monitoring, Diabetes self-management, and Overall knowledge. Each row contains data for Pre and Post intervention times. For General information about diabetic retinopathy, Pre Control is 99/1/0 and Post Control is 99/1/0, Pre Intervention is 100/0/0 and Post Intervention is 5/54/41. For Blood glucose monitoring, Pre Control is 61/32/7 and Post Control is 61/32/7, Pre Intervention is 81/17/2 and Post Intervention is 3/17/80. For Diabetes self-management, Pre Control is 99/1/-, and Post Control is 99/1/-, Pre Intervention is 100/0/-, and Post Intervention is 30/70/-. For Overall knowledge, Pre Control is 100/0/0 and Post Control is 100/0/0, Pre Intervention is 100/0/0 and Post Intervention is 10/34/56. The Chi-Square (p-Value) and Cramér’s V values are provided for each category.

Navigate back to Table 3..

## Table 4. Long description

The table has five rows and eight columns. The columns are labeled Domain, Time, Control n (%), Poor/Moderate/Good, Intervention n (%), Poor/Moderate/Good, Chi-squared (p-Value), and Cramér’s V. The rows are labeled Diet, Exercise, Medication adherence, Blood glucose monitoring, and Overall self-care. Each row shows the distribution of participants’ self-care practices before and after the intervention, with percentages and counts for poor, moderate, and good practices. The table also includes chi-squared values, p-values, and Cramér’s V for statistical significance.

Navigate back to Table 4..

## Table 5. Long description

The table presents data on changes in individual TPB constructs following an intervention. It has six rows and seven columns. The columns are labeled Construct, Group, Baseline mean ± SD, 3-month mean ± SD, Within-group change, Between-group difference (95% CI), p-Value, and Cohen’s d. The constructs listed are Attitude, Subjective norms, Perceived behavioural control, Behavioural intention, and Overall TPB. Each construct has data for both the Control and Intervention groups. For Attitude, the Control group shows a baseline mean of 29.8 ± 8.7 and a 3-month mean of 30.4 ± 9.2, with a within-group change of +0.6. The Intervention group shows a baseline mean of 30.7 ± 8.9 and a 3-month mean of 74.8 ± 10.6, with a within-group change of +44.1. The between-group difference is 44.4 (40.5-48.3) with a p-value of <0.001 and a Cohen’s d of 2.18. Similar data is presented for Subjective norms, Perceived behavioural control, Behavioural intention, and Overall TPB, showing substantial improvements in the Intervention group compared to negligible changes in the Control group.

Navigate back to Table 5..
