# Supplementary material for: Effect of a theory of planned behaviour-based self-care education programme on glycaemic control in patients with diabetic retinopathy: a quasi-experimental study
Source: J Nutr Sci. 2026 Jul 27;15:e67. doi: 10.1017/jns.2026.10133 (PMC13439020; doi:10.1017/jns.2026.10133)
Supplement: Supplementary file 1 [file S2048679026101335sup001.docx]

**Supplementary File 1** **English version of the 48-item questionnaire developed for the study** **“Factors Influencing Expatriates’ Preference for Direct versus Indirect Veneers in Erbil: The Role of Durability, Socioeconomic Status, and Patient Satisfaction: A Cross-Sectional Study”**

**Instructions:** Thank you for participating in this study. Please answer all questions honestly. There are no right or wrong answers. The questionnaire should take about 10-15 minutes to complete. All responses are confidential.

**Section 1: Socio-demographic Information (Items 1–9)**

1. What is your age? (in years) ________
2. What is your gender? ☐ Male ☐ Female ☐ Prefer not to say
3. What is your nationality / country of birth? ________________________
4. How long have you been living in Erbil as an expatriate? ☐ Less than 6 months ☐ 6–12 months ☐ 1–3 years ☐ More than 3 years
5. What is your highest level of education? ☐ Low (primary or below) ☐ Medium (secondary/high school) ☐ High (university degree or higher)
6. What is your current occupation? ________________________
7. How would you rate your current economic/financial status? ☐ Very poor ☐ Poor ☐ Medium ☐ Good ☐ Very good
8. Are you currently a student? ☐ Yes ☐ No
9. Have you ever received any cosmetic dental treatment before? ☐ Yes ☐ No

**Section 2: Prior Dental Experience and Information Sources (Items 10–18)**

10. Have you ever received dental veneers (direct or indirect)? ☐ Yes ☐ No
11. If yes, what type of veneers did you receive? (select all that apply) ☐ Direct composite veneers ☐ Indirect ceramic/porcelain veneers ☐ Both
12. How many veneers have you had in total? ________
13. Where did you first hear about dental veneers? (choose one main source) ☐ Dentist recommendation ☐ Social media (Instagram, etc.) ☐ Friends/family ☐ Internet search ☐ Television/magazine ☐ Other: ________
14. Have you had any previous dental restorations (fillings, crowns, etc.)? ☐ Yes ☐ No
15. How satisfied were you with your previous dental treatments overall? (1 = Very dissatisfied, 5 = Very satisfied) _____
16. Do you have regular dental check-ups? ☐ Yes (every 6 months) ☐ Occasionally ☐ Only when problem arises ☐ Never
17. Have you ever searched for information about direct vs indirect veneers? ☐ Yes ☐ No
18. Rate your current knowledge about the difference between direct and indirect veneers (1 = No knowledge, 5 = Excellent knowledge) _____

**Section 3: Decision-making Factors (Items 19–35)**

Please rate how important each of the following factors is to you when considering dental veneers. (5-point Likert scale: 1 = Not important at all, 2 = Slightly important, 3 = Moderately important, 4 = Important, 5 = Very important)

19. Aesthetic outcome / natural appearance of the smile
20. Durability / longevity of the veneers
21. Cost of the treatment
22. Treatment time / number of visits required
23. Minimally invasive preparation (how much tooth structure is removed)
24. Ease of repair if damaged
25. Resistance to staining / discoloration
26. Comfort during and after treatment
27. Reversibility of the procedure
28. Recommendation from the dentist
29. Influence of social media / celebrity smiles
30. Pain or sensitivity after treatment
31. Maintenance required (cleaning, polishing)
32. Possibility of achieving “Hollywood Smile”
33. Trust in the dental clinic / dentist’s experience
34. Availability of flexible payment plans
35. Long-term gum health around the veneers

**Section 4: Veneer-type Preference and Reasons (Items 36–41)**

36. Which type of veneer do you prefer? ☐ Direct composite veneers (done in one visit, chairside) ☐ Indirect veneers (ceramic/porcelain, made in lab) ☐ Undecided / need more information
37. If you prefer **indirect** veneers, what is the main reason? (choose one) ☐ Better durability ☐ Superior aesthetics ☐ Longer lasting ☐ Other: ________
38. If you prefer **direct** veneers, what is the main reason? (choose one) ☐ Lower cost ☐ Single visit / faster ☐ Less tooth reduction ☐ Other: ________
39. How much would you be willing to pay for indirect veneers compared to direct? (per tooth) (Open or options: e.g., Same price / 20% more / 50% more / etc.)
40. Would you choose indirect veneers if they last twice as long as direct ones? ☐ Yes ☐ No ☐ Maybe
41. What is the maximum number of dental visits you are willing to attend for veneer treatment? ________

**Section 5: Satisfaction and Recommendation (Items 42–48)**

(For those who have received veneers – if not, skip to general questions)

42. Overall, how satisfied are you with your veneers? (1 = Very dissatisfied … 5 = Very satisfied) _____
43. How satisfied are you with the **aesthetic** result? (1–5) _____
44. How satisfied are you with the **durability** so far? (1–5) _____
45. How satisfied are you with the **cost-effectiveness**? (1–5) _____
46. Would you recommend the same type of veneers to a friend or family member? ☐ Yes ☐ No ☐ Unsure
47. On a scale of 1–5, how likely are you to recommend veneers in general? (1 = Not at all likely … 5 = Extremely likely) _____
48. Any additional comments about your experience or preferences? (open text) ________________________

**End of Questionnaire** Thank you very much for your participation!
