# Supplementary material for: Effect of a theory of planned behaviour-based self-care education programme on glycaemic control in patients with diabetic retinopathy: a quasi-experimental study
Source: J Nutr Sci. 2026 Jul 27;15:e67. doi: 10.1017/jns.2026.10133 (PMC13439020; doi:10.1017/jns.2026.10133)
Supplement: Supplementary file 2 [file S2048679026101335sup002.docx]

**Structured health education program**

**Diabetic Retinopathy: keep your eyes on the glycemic control**

**Prepared by**

**Sazgar Ajeeb Mohammed**

PhD student in Adult Nursing

**Supervised**

**Dler Hamad Ismael**

B.Sc., M.Sc., PhD in Adult Nursing

**2024-2025**

**Topic: Diabetic Retinopathy: keep your eyes on the glycemic control**

**Place:** At Galyawa teaching center for diabetes & endocrinology in Erbil City

**Method of teaching:** Individual health education

**Teaching aids:** Booklet, pamphlet, questions and answers.

**Session schedule:** Three individual sessions, one per week during the first three weeks of Month 1, each of 20–35 minutes duration, at a fixed scheduled time at the Galyawa Teaching Center. Participants were contacted individually by WhatsApp 1–2 days before each session as a personal reminder.

**General objective**

To improve self-care education program for patients with diabetic retinopathy to glycemic control and health outcome to prevent further complications in the future of life.

**Specific objectives**

Enhance patient's knowledge and motivate them to practice through:

1-Understanding the meaning of self-care and disease itself concerning (predisposing factors, signs and symptoms, complications) and role of diet, exercise, monitoring blood glucose, using medication.

2- Following principle in monitoring blood glucose, insulin administration, choosing a healthy diet, exercising and using medication , blood pressure, lipid profile test, glycated hemoglobin and body mass index.

4- Enhance to change behavior of diabetic retinopathy patients to glycemic control by application of the Theory of Planned Behavior (TPB).

**Outline:**

- Describe theory planned behavior.
- Meaning of diabetes retinopathy.
- Signs and symptoms DR.
- Predisposing factors DR.
- Diagnostic tests of diabetes retinopathy.
- Management and treatments of diabetic retinopathy.

**Theory of Planned Behavior**

According this theory the researcher act to change behavior of DR patient to control HbA1c. The TPB posits that three cognitive constructs — attitude toward the behavior, subjective norms (perceived social influence), and perceived behavioral control — collectively determine behavioral intention, which in turn predicts actual behavior.

**Concept**

**Session 1 — Attitude and Knowledge**

The main goal was to improve patients’ understanding of diabetes retinopathy and control HbA1c. Participants were invited to attend knowledge and attitude, and we explained the objectives of the meeting to them. We emphasized the importance of understanding DR for successful self-management and provided a clear definition of DR, and discussed the role of insulin and medication in managing uncontrolled HbA1c. We also covered common symptoms and risk factors associated with DR. We also provided information on appropriate methods for checking blood sugar levels using a glucometer or continuous glucose monitor (CGM) and shared recommended target ranges for fasting and postprandial glucose levels. Additionally, we highlighted the role of exercise in controlling blood sugar levels and provided examples of suitable exercises that can be incorporated into daily routines based on individual preferences and limitations. We also discussed diet management techniques, including carbohydrate counting and the plate method approach, and offered practical tips on creating a balanced diet plan focusing on whole grains, proteins, vegetables/fruits/fiber-rich foods while limiting processed sugars, saturated fats, and alcohol intake. The session also focused on changing attitudes towards implementing these behaviors and addressing any obstacles that may hinder their adoption. Examples of potential complications associated with poor management were discussed, and a question-and-answer, a brainstorming activity was conducted where participants identified common obstacles or barriers to implementing self-management behaviors. In conclusion, key takeaways from the session highlighting the benefits of self-management behaviors in preventing complications were summarized.

**Session 2 — Subjective Norms**

During our second session, we aimed to evaluate subjective norm, and encourage dialogue about the social impact and perspectives of close individuals, family, doctors, ophthalmologist, dietitian healthcare workers on self-management behaviors. Our goal was to improve adherence to self-management behaviors by creating a supportive environment where participants could freely express their thoughts and feelings. and we followed a structured approach that focused on subjective norms and addressed the concept of social influence with self-management behaviors. We provided examples to illustrate how the opinions of close individuals, doctors, ophthalmologist, dietitian and healthcare workers can influence an individual’s commitment to self-management behaviors. Additionally, we encouraged participants to share their personal experiences with social influence related to their health.

**Session 3 — Perceived Behavioral Control and Behavioral Intention**

During the third session of our educational program, titled “Exploring Solutions to Facilitate Self-Management Behaviors,” our goal was to assist participants in learning strategies to make self-management behaviors easier. This involved teaching methods to facilitate exercise, measure blood sugar, insulin injection and teaching caregiver and develop positive attitudes toward implementing these behaviors. The session included Q&A sessions, and provide schedule to recommended target ranges for fasting and postprandial glucose levels, and education book. To enhance the learning process, we utilized educational aids such as clear pictures depicting successful patients who had effectively implemented self-management behaviors.

**Behavioral Intention:**

In intention focus on implementing and intention self-management behaviors and helping participants understand the consequences of their actions. Participants actively engaged in discussing the impacts of their behavior on self-management. They were encouraged to share personal experiences and insights, while also highlighting the positive outcomes that can be achieved by effectively managing one’s health. Emotional relief exercises were conducted, to help participants release any negative emotions or barriers they may have had towards adopting self-management behaviors. A speech was delivered emphasizing the importance of maintaining a positive attitude towards implementing self-management behaviors and highlighting the benefits that stem from this mindset. The main focus was to understand the reasons why self-management behaviors can be either beneficial or challenging. Various techniques were used to promote the adoption of these behaviors, such as question and answer sessions, brainstorming activities, and visual aids like images and education book.

**Understanding Diabetes**

In diabetes, the blood glucose level is too high either because the pancreas is not making enough insulin, or the insulin that is being made is not working correctly. Sometimes it is a combination of both.

**Diabetic Retinopathy:**

Diabetic retinopathy is caused by high blood sugar levels damaging the back of the eye retina. It can cause blindness if left undiagnosed and untreated (Sachdeva M., 2022).

**Symptoms of eye disease:**

• Blurred vision

• Dim vision

• Poor night vision

• Sensitivity to light

• Loss of vision

• Distorted vision

• Frequent eye glass script changes

Floating spots in your vision (Boyd *et al*., 2023).

**Risk Factors associated with Diabetic Retinopathy**

- All people with diabetes.

- Long diabetic duration.

- Poorly controlled blood sugar levels.

- High blood pressure.

- High blood cholesterol.

-Pregnancy.

- Obesity.

- Tobacco smokers (Fariza .,2017).

**Diagnostic test:**

**1- Glycated hemoglobin (HbA1c):**

The HbA1c is a lab test that shows the average blood glucose level in the past 8-12 weeks should be less than 7%. It is the best way to know overall blood glucose control.

- Consistently high levels of HbA1c mean a greater risk of development and progression of diabetes-related retinopathy,. Improving diabetes control by decreasing HbA1c reduces chance of developing diabetes-related retinopathy and can reverse diabetes eye changes that already exist.

- Can improve A1c through meal planning, exercise, medication, monitoring, and managing stress (Bin Rakhis *et al*.,2022).

**Red blood cells A1c number**

-Healthy red blood cells have some glucose attached to them.

-A high A1c means that too much glucose is attached to the cells.

**2-Visual acuity test:**

**This eye chart test measures how well see at various distances.**

**3-Dilated Eye Exam**:

 Is best diagnosed because there are no warning signs for diabetic retinopathy. A dilated eye exam allows the eye care professional to see the early signs of the disease and help before vision is affected. Finding and treating diabetic retinopathy early can help protect vision. For this exam, drops placed in eyes widen (dilate) pupils to allow doctor a better view inside eyes. The drops can cause close vision to blur until they wear off, several hours later **(NEHEP.,2020).**

**Management of diabetic retinopathy**

**1- Self-monitoring blood glucose level (SMBG)**

Self-monitoring of blood glucose: refers to home blood glucose testing for people with type-2 diabetes, People with type-2 diabetes are responsible for the management of their diabetes through a program of self-care including maintaining a healthy lifestyle; self-monitoring of blood glucose.

Target blood glucose levels for People with diabetes before meals 70 to 130 and two hours after starting off the meal should be less than 180.

**Commonly recommended blood glucose monitoring plan**

- Before meals and before taking insulin or medication.
- Two hours after meals.
- Before bedtime.

**Steps for monitoring blood glucose level**

1. Hand washing

2. Put the test strip in the meter

3. Prick (stick) finger by lancet and squeeze of figure

4. Touch the drop of blood of finger to the test strip

5. Record the result of the test (Cafasso.,2022)

**2-Controling blood pressure**

- Keeping blood pressure in check can prevent or delay progression of diabetic retinopathy.

. Ideal blood pressure is 120/80 mm Hg. It is detected by check up only.

- It is treated by lifestyle changes and medication control blood pressure.

- Use less salt, be physically active, and do exercise like brisk walking, aerobics for 30 minutes.

**Some tips to lower your sodium (salt) intake:**

- **Look** for foods that are labeled “low-sodium,” “sodium-free,” “no salt added,” or “unsalted.” Check the total sodium content on food labels.
- **Don’t** cook with salt or add salt to your food. Try pepper, garlic, lemon, or other spices for flavor instead.
- **Avoid** processed meats (particularly cured meats, bacon, hot dogs, sausage ). Processed meats have been associated with increased risk for heart disease.
- **Avoid** foods that are naturally high in sodium, like, nuts, olives, soy tomato and other vegetable juices, and cheese.
- **Take** care when eating out. Stick to steamed, grilled, baked, boiled, and broiled foods with no added salt, sauce, or cheese.
- **Use** oil and vinegar, rather than bottled dressings, on salads.
- **Eat** fresh fruit or sorbet when having dessert.

**3- Controlling lipid profile**

Cholesterol is fat-like substance your body produces naturally. It's in the bloodstream and in your body’s cells. Triglycerides and Low-density

lipoprotein (LDL) is often called the “bad” kind. When you have too much LDL cholesterol in your blood, it can join with fats and other substances to build up in the inner walls of your arteries, creating a thick, hard substance called plaque. The “good” kind of cholesterol is high-density lipoprotein (HDL). It removes the “bad” LDL cholesterol away from the arteries and back to the liver, so it can be removed from your body. (**Puigdollers *et al* ., 2018**).

**4- Eating a healthy balanced diet**

**Goals of healthy diet**

1. To promote and support healthful eating patterns, emphasizing a variety of nutrient-dense foods in appropriate portion sizes, in order to improve overall health and specifically to:

- Achieve and maintain body weight goals

- Attain individualized glycemic, blood pressure, and lipid goals

- Delay or prevent the complications of diabetes (American Diabetes Association, 2017).

**Carbohydrate:**

***Recommended:*** Whole grain quinoa., black rice, brown rice. Vegetables and fruits (Blue berries, Avocado, carrots, pumpkin, spinach ,broccoli, , Orange and kiwi ), legumes with control portion such as (Baked beans, lentils), and minimally processed grains.

***Not Recommended:*** High sugar content, refined carbohydrates or processed grains and starchy foods especially sugary beverages, most pasta, white bread, white rice, low-fiber cereal, and white potatoes should be consumed in limited quantities.

**Fat:**

***Recommended:*** Mono and polyunsaturated fats (e.g., olive oil, and fish, nuts/seeds, particularly those high in omega-3 fatty acids). oily fish (e.g., salmon , tuna , sardines) 2 times/week, as a source of omega-3 fatty acids.

***Not Recommended:*** Foods high in saturated fat, including beef, lamb and high-fat dairy products (e.g., whole milk or yogurt, cream cheese) Foods high in trans-fats (e.g., fast foods, commercially baked goods, some margarine). Foods high in dietary cholesterol such as organ meats and egg yolks.

**Protein:**

***Recommended:*** Fish, skinless poultry, non-fat or low-fat dairy, nuts, seeds, and legumes

***Not Recommended:*** High saturated fat protein sources in excess (e.g., beef, lamb and high-fat dairy products).

**Plate method**

-The plate method External link helps control portion sizes without counting and measuring. This method divides a 9-inch plate into the following three sections to help choose the types and amounts of foods External link to eat for each meal.

- Nonstarchy vegetables—such as leafy greens, peppers, carrots, or green beans—should make up half of plate.

- Carb foods that are high in fiber—such as brown rice, whole grains, beans, or fruits—should make up one-quarter of plate.

- Protein foods—such as lean meats, fish, dairy, or tofu or other soy products—should make up one quarter of your plate.

- If you are not taking insulin, you may not need to count carbs when using the plate method.

Plate method, with half of the circular plate filled with nonstarchy vegetables; one fourth of the plate showing carbohydrate foods, including fruits; and one fourth of the plate showing protein foods. A glass filled with water, or another zero-calorie drink, is on the side.( NIDDK, 2023).

**The following charts highlight low, medium, and high GI foods**

| **Food Group** \| **Low GI (1-55)** \| **Medium GI (56-69)** \| **High GI (70-100)** | **Food Group** \| **Low GI (1-55)** \| **Medium GI (56-69)** \| **High GI (70-100)** | **Food Group** \| **Low GI (1-55)** \| **Medium GI (56-69)** \| **High GI (70-100)** | **Food Group** \| **Low GI (1-55)** \| **Medium GI (56-69)** \| **High GI (70-100)** |
| --- | --- | --- | --- |
| **Vegetable**   | Eggplant | Corn | Potatoes |
|  | Garlic | Leeks |  |
|  | Carrots | Beets |  |
|  | Green peas |  |  |
|  | Onions |  |  |
|  | Avocados |  |  |
|  | Peppers |  |  |
|  | Broccoli |  |  |
|  | Cabbage |  |  |
|  | Cauliflower |  |  |
|  | Celery |  |  |
|  | Cucumbers |  |  |
|  | Green beans |  |  |
|  | Mushrooms |  |  |
|  | Tomatoes |  |  |
|  | Lettuce |  |  |
|  | Squash |  |  |
|  | Spinach |  |  |
|  | Onion |  |  |
| **Fruits**   | Cherries | Apricots | Dates |
|  | Apples | Cantaloupe | Watermelon |
|  | Mangoes | Banana | Melon |
|  | pears | Pineapple |  |
|  | Grapes, Green | Figs |  |
|  | Grapes | Pears |  |
|  | Kiwi | Apricots |  |
|  | Lemons/Limes |  |  |
|  | Oranges |  |  |
|  | Plums |  |  |
|  | Strawberry |  |  |
| **Nuts & Seeds**   | Pistachio |  |  |
|  | Walnuts |  |  |
|  | Sunflower seeds |  |  |
|  | Pumpkin seeds |  |  |
|  | Peanuts |  |  |
|  | Almonds |  |  |
|  | Sesame seeds |  |  |
| **Beans & Leg**   | Chickpea |  |  |
|  | Kidney beans |  |  |
|  | lentils |  |  |
|  | Bean |  |  |
| **Sugar and Sweeteners**   |  | Brown sugar | White sugar |
|  |  |  | Honey |
| **Grains**   | Oats |  | White Rice |
|  | Barley |  | White Bread |
|  | Brown rice |  |  |
| **Drinks**   | Water | Fanta |  |
|  |  | Coca-cola |  |

**5- Physical activity:**

General Exercise Recommendations:

For good health, try to build up to 210 minutes per week, or 30 minutes every day of moderate intensity physical activity. You can break down exercise bouts into smaller 10 minute intervals if 30 minutes is too long at once.

**Benefits include:**

- Weight loss
- Lower blood pressure
- Lower cholesterol
- Increased strength
- Better rest
- An increased sense of wellbeing.

• **Aerobic physical activity:** such as walking, jogging,

swimming, cycling or dancing – any activities that get

the large muscle groups moving and are repetitive.

• **Resistance exercises (muscle strengthening):** targeting all of the major muscle groups (arms, legs, chest, back and abdominal) resistance exercises are those that cause the muscles to move against a force such as weight or gravity.

• **Incidental or unstructured activities** - try to limit or break up the time spent sitting and find ways to move more such as taking the stairs and parking further away. When completing resistance training exercise aim to perform:

• 8-10 different exercises.

• 2-3 sets of 8-10 repetitions. When starting out, perform the exercises with little or no weight.

As you get stronger and more confident, work your way up to a weight that is tough to lift on the 10th time. • 2-3 days of the week.

Exercising Precautions

• High impact exercise Excessive leaping and

jumping can further harm already damaged tissue.

• Holding breath and straining heavily can cause your

blood pressure to increase unnecessarily. This can cause

damage to the small blood vessels in the eye when

pressure is sustained.

• Exercising with head below the level of heart: This causes a

large increase in blood pressure and pressure within the eyes

(intraocular pressure). This can cause the tiny eye blood vessels

to rupture in those with severe retinopathy. Other activities that

can increase “intraocular” pressure are scuba diving, skydiving

and even some yoga and Pilates activities (ADC., 2011).

**6- Diabetic medications**

• Different medications work in different ways and have different forms

• You provider may prescribe Insulin or a non-insulin medication

• Multiple medications may be required to treat your diabetes.

• Medications work best with a healthy eating and staying active.

**Oral diabetes medication**

How Biguanide works in the body.

• It decreases glucose released from the liver.

• It reduces cardiac risks.

• It makes tissue more sensitive to insulin.

There are some possible side effects:

• Nausea

• Flatulence

• Diarrhea

TIPS on taking this medicine:

• Take with food to reduce side effects.

• Begin with lower dose and increase slowly.

• Talk to your Provider if having a test with contrast dye.

**Insulin:**

• There are different kinds of insulin. Some act over a full day and others give a quick burst to match what you are eating.

• Long-acting insulin (e.g., Lantus®) works all day long but is generally not enough to control blood glucose when you eat.

• Therefore, you usually do not skip this insulin if you have to skip a meal.

• Long-acting insulin should be taken at the same time every day.

• Quick-acting insulin (e.g., NovoLog® or Regular) gets into your blood stream quickly and lasts for a few hours.

• Take NovoLog® 5-15 minutes before your meal or take Regular 30 minutes before.

• If you skip or delay a meal, you should skip or delay your quick-acting insulin as well.

• Quick-acting insulin can also be used to quickly bring down high blood glucose, called “correction insulin”.

**Site of insulin injection**

-Insulin injections are administrated into the

subcutaneous tissue with the use of special

insulin syringes. The four main areas for

injections are the abdomen, arms, thigh,

and hips (abdomen and thigh areas are convenient

for self-administration of insulin) insulin usually

needs to be administered half hour before mealtimes.

**How to self- administration of insulin**

**Follow these steps for safe and proper injection:**

1-Wash hands with warm, soapy water

2-If using cloudy insulin, roll bottle between hands.

3-Clean rubber stopper of insulin bottle with alcohol

4-Fill syringe with air equal to the number of units of insulin you will be taking.

5. Insert the needle into the bottle and push air into the insulin bottle

6-Draw syringe and bottle upside down and draw out dose of insulin.

7-Gently pinch a 1- to the 2-inch portion of skin. And insert the needle at a 45-degree angle. Push the plunger all the way down and wait for 10 seconds. (Morris S.Y., 2023).

**7- Maintaining a healthy weight**

Excess weight will affect blood pressure, blood cholesterol

and blood glucose levels. If are overweight, reducing your

weight by just 5-10% can have significant health benefits.

• Extra weight can cause insulin resistance. Losing weight

could help insulin you produce or inject work properly.

• Even a 5% loss of body weight can improve your insulin resistance.

• If you are overweight, focus on eating fewer calories or weight loss of 3-5%.

• For healthy weight, decrease fat intake.

• May result in taking less medication. ( Furey and Edward., 2023)**.**

**Treatment of Diabetes Retinopathy:**

**1.Early diabetic retinopathy**:

If have mild or moderate non proliferative diabetic retinopathy, might not need treatment right away.

**2.Advanced diabetic retinopathy**:

If have proliferative diabetic retinopathy or macular edema, need prompt treatment. Depending on the specific problems with retina, options might include:

**a.Photocoagulation (laser treatment):**

This laser treatment, also known as focal laser treatment,

can stop or slow the leakage of blood and fluid in the eye.

During the procedure, leaks from abnormal blood vessels

are treated with laser burns.

**b. Anti- VEGF (vascular endothelial growth factor) drug:**

Injecting medications into the vitreous of the eye. They help

stop growth of new blood vessels and decrease fluid buildup.

**c. Vitrectomy:**

 This procedure uses a tiny incision in eye to remove blood

from the middle of the eye (vitreous) as well as scar tissue

that's tugging on the retina. It's done in a surgery center or

hospital using local or general anesthesia. **The procedure is**

**used to treat severe bleeding into the vitreous (NEI.,2020).**

**References:**

American Diabetes Association., (2016). Lifestyle Management. Diabetes Care .[Internet]. [Cited 2024 June 04].40(1), S33–S43 Available from: https://doi.org/10.2337/dc17-S007

Austuralian Diabetic Councel .,(2011).diabetic and eye health. [Internet]. [Cited 2024 July 30].Avialable from:https://www.eesc.com.au/new/wp-content/uploads/Diabetes-and-Eye-Booklet-DAW-2011.pdf.p-p.18-19.

Bell ,A.M.,( 2020). Hypoglycemic Episode: Signs, Prevention, and Action Steps. [Internet]. [Cited 2024 July 29] . Available from: https://www.healthline.com/health/diabetes/hypoglycemic-action-plan.

Bin Rakhis, S.A., AlDuwayhis, N.M., Aleid, N., AlBarrak, A.N., Aloraini, A.A., (2022). Glycemic Control for Type 2 Diabetes Mellitus Patients: A Systematic Review. [Internet].[Cited 2024 July 11].14( 6), 26180. Available from: <https://www.ncbi.nlm.nih.gov/pmc/articles/PMC9304683/>

<https://doi.org/10.7759/cureus.26180>

British Dietetic Association., (2016). Food Fact Sheet is for people with Type 2 Diabetes .[Internet]. [Cited 2024 July 28].Available from: <https://www.bda.uk.com/foodfacts.accessed%20by%202014%20July%2028>

[Boyd](https://www.aao.org/biography/eac93724-3619-4834-8f38-50520c476300), K., Vemulakonda, G.A., Turbert, D., ( 2023). Diabetic retinopathy: Causes, symptoms, and treatments. .[Internet].[Cited 2024 July 3]. Available from: <https://www.medicalnewstoday.com/articles/183417.>

Cafasso, J .,(2022).A Guide to Blood Glucose Tests: Preparation and Results [Internet], [Cited 2024 July31]. Available from: URL https://www.healthline.com/health/glucose-test-blood.

Etheridge J.C and Brin+dle M.E.,(2023).Theory of Planned Behavior - an overview .Science Direct Topics. [Internet], [Cited 2024 August 5]. 1 ( 4 ), 0749 - 0712. Available from: https://www.sciencedirect.com/topics/medicine-and-dentistry/theory-of-planned-behavior .

Fatih, N.D. (2024). The 4 Stages of Diabetic Retinopathy . [Cited 2024 May 23]. Available from: https://www.drsnyder.org/eye-care-services/eye-disease-management/diabetic-retinopathy/the-4-stages-of-diabetic-retinopathy/ .

Ferrucci S., (2019). Standard Tools and Tests for Diagnosing Diabetic Retinopathy. [Internet].[cited 2024 July 5]. Mod. Optom. Available from: https://modernod.com/articles/2019-june/standard-tools-and-tests-fordiagnosing-diabetic-retinopathy.

Furey and Edward., (2023). BMI Calculator Body Mass Index [Internet]. [cited 2024 July 29]. Available from: URL <https://www.calculatorsoup.com/calculators/health/bmi-calculator.php>.

Fariza N, Zahirah N, Zakiah Z, Anita N, Marina M., 2017. Diabetic retinopathy screening.[Internet].[cited 2024 July 12]. Available from:? [moh.gov.my/moh/resources/Penerbitan/2017/Rujukan/NCD 2017/Diabetic_Retinopathy_Screening_Module.pdf](https://www.moh.gov.my/moh/resources/Penerbitan/2017/Rujukan/NCD%202017/Diabetic_Retinopathy_Screening_Module.pdf)

Hall, C.E., Hall, A.B., Mallya, J., Courtright, P., Kok, G., 2022. Establishing a screening programme for diabetic retinopathy in Kilimanjaro Region, Tanzania using intervention mapping. Eye .[Internet].[Cited 2024 July 23]. 36(1), 17–24. Available from:? <https://doi.org/10.1038/s41433-022-02001-w.>

LaMorte W.W .,(2022).The Theory of Planned Behavior . [Internet], [Cited 2024 August 5]. Available from: URL https://sphweb.bumc.bu.edu/otlt/mph-modules/sb/behavioralchangetheories/BehavioralChangeTheories3.html (accessed 8.5.24).

Morris S.Y., (2023).Insulin Injection Sites: Where and How to Inject [Internet]. [Cited 2024 July 30]. Available from: URL https://www.healthline.com/health/diabetes/insulin-injection

Mcloed.,(2022).The Theory of Planned Behavior: Behavioral Intention. [Internet], [Cited 2024 August 5]. Available from: URL https://www.simplypsychology.org/theory-of-planned-behavior.html.

Morgan, K.K.,( 2024). How to Use the Glycemic Index . [Internet], [Cited 2024 August 11]. Available from: URL https://www.webmd.com/diabetes/glycemic-index-good-versus-bad-carbs.

National Health Institute of Diabetes and Diegestive and Kidney Disease., 2023. Healthy Living with Diabetes - NIDDK [Internet], [cited 2024 August 30]. Available from:. URL https://www.niddk.nih.gov/health-information/diabetes/overview/healthy-living-with-diabetes.

National Eye Institute (2020). Diabetic retinopathy. Accessed by: 2024 May 24. P.24. Available from: [610184772de0bb06e1516895_diabeticretinopathy-information.pdf (website-files.com)](https://assets.website-files.com/610184772de0bb58435167bf/610184772de0bb06e1516895_diabeticretinopathy-information.pdf)

National Eye Health Education Program(2020).diabetic eye disease [internet]. [Cited 2024 July 29]. Available from: https://www.nei.nih.gov/sites/default/files/2023-02/Diabetic_Eye_Disease_Educators_Guide.pdf.

Paul, A.J., (2021). Advances in Classifying the Stages of Diabetic Retinopathy Using Convolutional Neural Networks in Low Memory Edge Devices.[Internet].[Cited 2024 July 5]. Available from:

https://www.drsnyder.org/eye-care-services/eye-disease-management/diabetic-retinopathy/the-4-stages-

URLhttps://webeye.ophth.uiowa.edu/eyeforum/tutorials/diabetic-retinopathy-med-students/Classification.htm.

**Puigdollers A .S., Roca M.F., Grima V.H.,(2018).Causes of Diabetic Retinopathy | Hospital Clínic Barcelona.[Internet].[Cited 2024 July 3].** **Available from: https://www.clinicbarcelona.org/en/assistance/diseases/diabetic-retinopathy/causes-and-risk-factors.**

Services Department of Health ., (2024). Carbohydrates and the glycaemic index. [Internet], [Cited 2024 August 11]. Available from: http://www.betterhealth.vic.gov.au/health/healthyliving/carbohydrates-and-the-glycaemic-index

Sachdeva M., (2022). Diabetic Retinopathy. [Internet], [cited 2024 August 23]. Available from: <https://www.hopkinsmedicine.org/health/conditions-and-diseases/diabetes/diabetic-retinopa>thy.

Wang, W., Lo, A.C.Y., (2018). Diabetic Retinopathy: Pathophysiology and Treatments .Int J Mol Sci.[Internet].[Cited 2024 July 4].19(6),1816 . Available from: DOI:10.3390/ijms19061816.

Wolfensberger, T.J.,and Hamilton, A.M.P., 2001. Diabetic retinopathy – An historical review. Semin. Ophthalmol.[Internet].[Cited 2024 July 1].16(1),2-7.Available from: https://www.tandfonline.com/doi/abs/10.1076/soph.16.1.2.4220.
